# Miso: A traditional nutritious & health‐endorsing fermented product

**DOI:** 10.1002/fsn3.3029

**Published:** 2022-09-15

**Authors:** Farhan Saeed, Muhammad Afzaal, Yasir Abbas Shah, Mujahid Hassan Khan, Muzzamal Hussain, Ali Ikram, Huda Ateeq, Muhammad Noman, Shamaail A. Saewan, Ashraf O. Khashroum

**Affiliations:** ^1^ Department of Food Sciences Government College University Faisalabad Pakistan; ^2^ Department of Food Technology Chulalongkorn University Bangkok Thailand; ^3^ University Institute of Food Science and Technology, The University of Lahore Lahore Pakistan; ^4^ Department of Food Sciences College of Agriculture, University of Basrah Basrah Iraq; ^5^ Department of Plant Production and Protection, Faculty of Agriculture Jerash University Jerash Jordan

**Keywords:** enzymes, fermentation, microbial community, miso, nutritional composition, therapeutic potential

## Abstract

Consumer demand for fermented foods with a well‐balanced nutrient profile has been increasing owing to their ability to prevent chronic diseases as well as their functional, nutritional, and nutraceutical benefits. Among those functional foods, miso is a well‐known traditional fermented food with a distinctive savory flavor and aroma that is most commonly used as a seasoning in miso soup. Among different fermented products, miso is derived from soybeans and grains as a result of the activities of *Koji* enzymes and beneficial microbes. Additionally, the microbial community of miso is thought to be crucial in enhancing its distinct flavor and texture as well as its nutritional properties. Despite the importance of microorganisms in the production of miso, there has been relatively little research done to characterize and describe the nutritional and medicinal potential of miso. In this review, the potential therapeutic properties, i.e., anticancer, antimicrobial, and antiobesity, of miso have been discussed comprehensively. This review envisions the production technology, its history, microbial population, nutritional properties, and the potential health benefits of miso associated with its consumption.

## Graphical abstract

Miso is a well‐known traditional fermented food. In addition, this review envisions the production technology, its history, microbial population, nutritional properties and the potential health benefits of miso associated with its consumptions. In this review, the potential therapeutic properties i.e., anti‐cancer, anti‐microbial, anti‐obesity of miso have been discussed comprehensively. Furthermore, the positive effects of miso consumption, eliminating the radioactive effects, and regulating the cholesterol levels in the human body, are the limelight of this article.

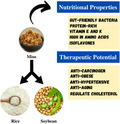

## 1 OVERVIEW

Fermentation has been used to modify and produce foods since antiquity. Humans gradually discovered that the qualities of foods changed while they were stored and that some of these changes provided desirable new flavor profiles and preserve the food (Allwood et al., 2021). Fermentation produces a wide range of preserved foods, which are becoming widely popular. Fermented soybean products, such as cheonggukjang (Japanese natto), doenjang (soy paste), ganjang (soy sauce), and douchi, are widely consumed in East Asian countries and are major sources of bioactive compounds. The fermentation of cooked soybean with bacteria (*Bacillus* spp.) and fungi (*Aspergillus* spp. and *Rhizopus* spp.) produces a variety of novel compounds, most of which possess health benefits.

Miso is one of the fundamental seasonings used in Japanese cuisine made by the fermentation of soybean paste. Miso is among the conventional and distinctive fermentation products (Farnworth, 2008). Soybean fermented products are used by the Japanese community since ancient times. Fermented soybean products are a vital part of their dinner table. Miso is a rice‐based food product so it is inexpensive and easily available as it is cultivated in Japan for very long. Soy sauce has a resembling taste of miso. Miso is formed by combining two fermentation processes. To prepare *Koji*, a substrate is first inoculated with a mold, usually, *Aspergillus oryzae*, and then fermented. After that, the *Koji* is mixed with salt and soybean mash and fermented again, this time by yeast and bacteria (Allwood et al., 2021). During fermentation, the microbial community of *Koji* and miso is thought to be crucial to the formation of its unique flavor, texture, and nutritional composition (Dimidi et al., 2019). Despite the importance of microorganisms in miso manufacturing, little research has been done to describe their populations and processes. There is a long history of Japanese people growing soybeans and discovering different and innovative seasonings and food products. The distinctive savory flavor of miso is becoming very popular in Western cuisine, where it is used as a seasoning in marinades, and even in sweets by cooks and chefs. Miso is also used in dressings, marinades, stews, casseroles, and dips (Watanabe, 2013).

**FIGURE 1:**
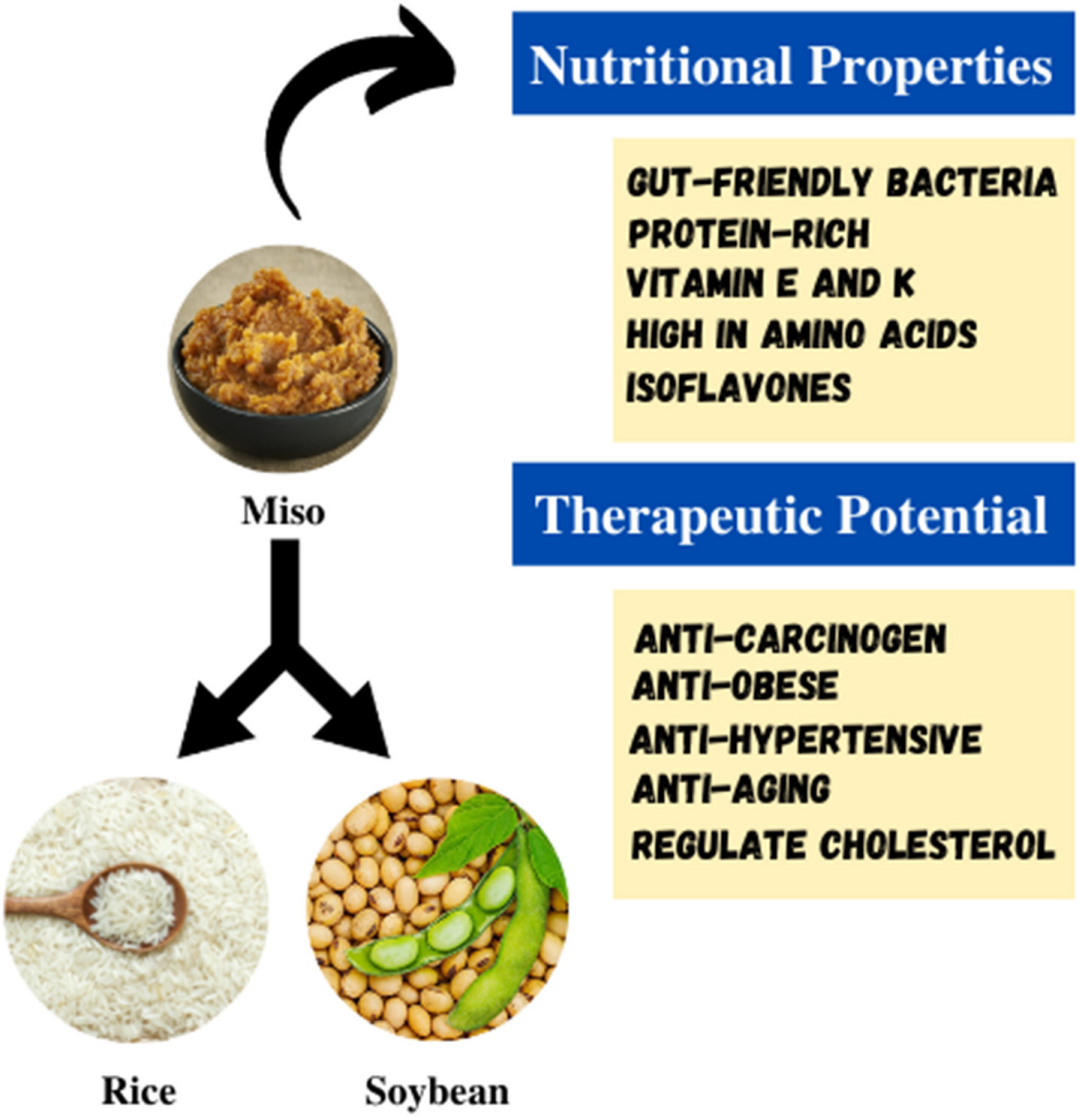
Graphical abstract

Miso is classified into four categories based on the ingredients used: rice, barley, soybean, and mixed miso. Rice miso is made from rice, soybeans, and salt. Rice is fermented with *Koji* mold to make *Koji*, which is then used for soybean fermentation and maturation. Barley miso is prepared in the same method as rice miso, except instead of rice, barley, or naked barley is used. Soybean miso is prepared from soybeans and salt, and it is fermented and aged with soybean *Koji*. Mixed miso can be made with a mixture of rice, barley, and/or soybean *Koji* or any miso made with a mixture of rice, barley, and/or soybean *Koji* (Kusumoto et al., 2021). Miso includes vitamins, minerals, vegetable proteins, microorganisms, salts, carbohydrates, and fat (Watanabe et al., 2006). This fermented product not only has a distinct mouthfeel and flavor but it is also related to several health benefits, including ant oxidative properties, improved digestion, and a stronger immune system (Jayachandran & Xu, 2019). This fermented product contains bioactive compounds and various nutrients and it has potential therapeutical properties, also shown in Figure 1. Various studies have revealed the therapeutic potential of miso. Miso is now known as a functional food as a result of these properties, and its popularity is rapidly increasing all around the world. This review is focused to summarize the nutritional properties and various health benefits of miso.

## 2 HISTORICAL BACKGROUND

The origin of miso is thought to be in China around 600 A.D. or perhaps sooner. In Japan, it is called miso; in China, it is called Chiang; in Indonesia, tauco; in Korea, Jang or doenjang; in Thailand, taochieo; and in the Philippines, tao‐si. The majority of these items contain *Koji*, which is made from rice or barley fermented by *Aspergillus oryzae* (Farnworth, 2008). Chinese food is known as “chiang,” which was Miso's earliest known ancestor, which is believed to have arisen before the Chau dynasty (722–481 BC). The earliest assortments of chiang were made with fish, shellfish, and game, according to Shurtleff and Aoyagi (1976). Miso did not become common until the fourth century AD, sometime between the second and third centuries BC. It is believed to have been brought to Japan shortly before Buddhism was introduced (540–552 AD). “Miso was a fermented food with soybeans as its main ingredient, but also containing corn, rice *Koji*, wheat, salt, and saki,” according to the book (Shurtleff & Aoyagi, 1976). According to these authors, Otto Kellner, a German, was the first westerner to research the miso‐making method, publishing a comprehensive article on the subject in 1893.

“The Soybean,” which offers valuable knowledge about miso was written in the classical work of Piper and Morse (1923), “Miso is said to exceed all other soybean preparations in the Orient in terms of use,” they write. There is evidence that the fermentation of miso precedes the development of soy sauce (Abiose et al., 1982). Soy sauce was originally known as taimari miso, according to these workers. Tamari, which translates to “liquid drip,” is the name given to the liquid that drips to the bottom of a miso keg as it ages. As a result, miso was used to produce tamari, Japan's original form of soy sauce. In reality, miso is said to be Japan's oldest fermented food (Abiose et al., 1982; Shurtleff & Aoyagi, 1976). In Japan, there are now a large number of miso factories (Johlas et al., 2020).

Miso, like wine and cheese in Western societies, is a significant aspect of cuisine culture that represents the feudal unique customs of Japan. Although miso comes in a variety of forms, the most widespread usage in foods is in miso soup, a side dish with a variety of seasonal vegetables or locally sourced ingredients (Tamang et al., 2021). Miso is made both locally and on a large scale in Japan, and it has one of the longest histories in the food industry. Fermentation processes, production procedure standardization, and quality controls are all well established. Miso consumption has been linked to health benefits like lower risk of mortality and the incidence of certain diseases.

## 3 PRODUCTION TECHNOLOGY

Different types of miso are manufactured by just making differences in their constituent ratio, time of fermentation (aging), and other parameters. *Koji* mold is a fungus used for fermentation. *Aspergillus oryzae* is a yellow‐colored mold that is used for the process of brewing specifically in Japan. Practically, the enzymes (specifically amylases) present in *Koji* help to convert the rice starch to sugar. Miso classification is based on the ingredients used in the manufacture of the product. The most commonly known type of miso is rice miso which is made with rice (*Koji*) starter (Moriyama et al., 2013).

*Koji* or steamed rice (barley) inoculated with *Aspergillus oryzae* for 40–48 h is used as a starter for the fermentation process in the formation of miso. Soybeans are soaked overnight in water and then pressure cooked till they are soft. After that, the beans are mashed and kneaded along with *Koji*, salt, and water. The paste is then packed into a sealed glass jar and kept in a cool, dark place to ferment at a temperature of 25–30°C (Chan et al., 2020). Soybeans, rice, salt, and *Koji* are some of the main components in the production of miso. Although there are many different types of soybeans for miso manufacturing, only a few cultivars are of high enough quality to be utilized for processing in Japan. Because magnesium and calcium are beneficial to the fermentation process and have a positive influence on it, seawater salt is advised for miso manufacturing. In recent years, many Japanese institutes have begun research into determining and selecting the best miso *Koji* strain, as well as standardizing miso products in this way. Fungus strains are accessible in Japan via the internet at miso stores (Wang et al., 2019).

**FIGURE 2:**
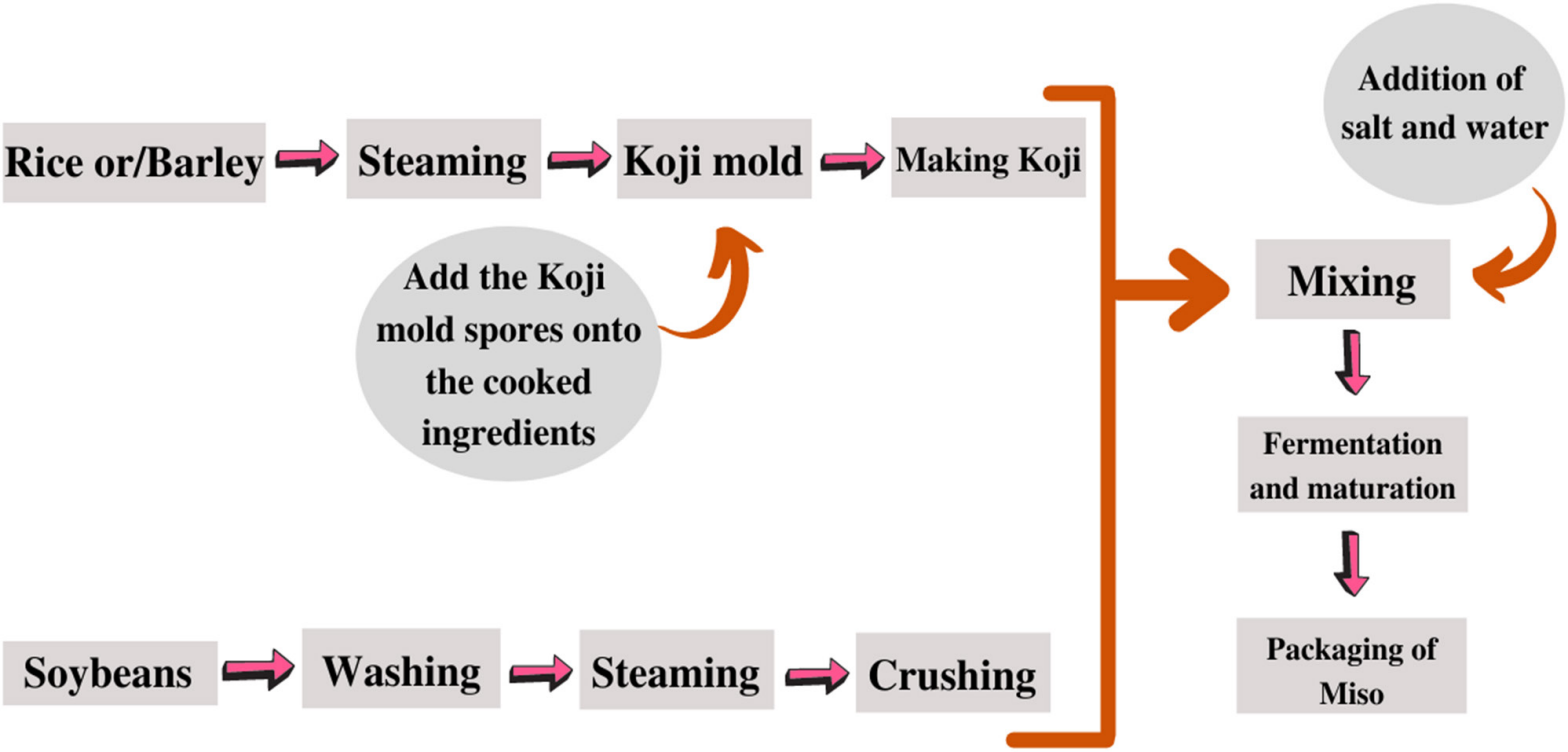
Steps in miso production

Other forms of miso include barley miso and soy miso, in addition to rice miso (dark brown, brown, cream, or white with varying salt content) (Ohata et al., 2009). Rice miso accounts for over 80% of miso produced in Japan, with dark‐brown miso being the most popular. Miso paste, which is commonly used to make soup, can also be used as a sauce for the marinade, dressing, and dipping sauce. Figure 2 represents the steps involved in miso production.

## 4 MICROBIOLOGY

Microbes play a role in changing the nutritional quality of foods in a variety of ways, particularly those involved in processes such as fermentation. This may increase the bioavailability of nutrients in the diet. Alternatively, the fermentation of bacteria can sometimes produce new components that are nutritionally useful to the body. Microorganisms may produce their cellular enzymes or other physiologically active compounds. These enzymes/active chemicals are consumed along with the fermented meals and perform vital functions in the body of the host. Probiotics are foods that contain bacteria that have been determined to be beneficial to one's health. Functional foods are described as foods that are similar in appearance to regular foods but provide health benefits and/or reduce the risk of chronic illness beyond the fundamental nutrients that food delivers. Probiotics are one type of functional food that contain bacteria that have been determined to be beneficial to one's health.

Two studies examined the microbial population in Japanese miso that had been purchased. In a 2003 study, researchers employed traditional culture and biochemical techniques to determine the bacterial communities in a single batch of miso prepared in Japan (Onda et al., 2003). The most prominent LAB in the final miso product were *T. halophilus* and the nonhalophilic (moderately salt‐tolerant) *Enterococcus faecium* species. *E. faecium* species may be significant for miso fermentation because the bacteriocins produced by this species have been reported to have antibacterial activity against spoilage bacteria in miso (Onda et al., 2003). Another research, conducted in 2010, used nested polymerase chain reaction‐denaturing gradient gel electrophoresis (PCR‐DGGE) to examine five purchased Japanese soybean pastes (Kim et al., 2010). The predominant fungal species detected in the miso samples were *A. oryzae* and the yeast *Z. rouxii*, according to the findings. The two most common bacterial species were *T. halophilis* and *S. gallinarum*. However, the study did not give details on the length of fermentation, ingredients, manufacturer, storage before purchase, or use‐by dates for these samples.

The culture‐dependent methods used in these studies have a limitation in that they do not recognize unculturable bacteria and hence may not show the entire complement of microorganisms present. More research is needed to examine the microbial population of miso. Common fermented foods of soybean origin are described with their microbial composition in Table 1.

**TABLE 1:** Microbial composition of soybean‐origin‐fermented food products

Name	Description	Origin Regions	Source of Microorganisms	Microbes Identified in Final Product
Miso	Fermented soybean pastes	Japan	Starter culture (*Aspergillus oryzae*)	*Bacillus amyloliquefaciens*, *Bacillus subtilis*, *Staphylococcus kloosii*, *Staphylococcus gallinarum*, and *Lactococcus* sp. GM005
Natto	Fermented soybean	Japan	Starter culture (*Bacillus subtilis* natto)	*Lactobacillus plantarum* TK9 and *Lactobacillus paracasei* TK1501
Tempeh	Fermented boiled and dehulled soybeans	Indonesia	Starter culture (*Rhizopus oligoporus*)	*Rhizopus oryzae*, *Mucor indicus*, *Geotrichum candidum*, *Alternaria alternata*, *Trichosporon beigelii*, *Candida maltosa*, *Yarrowia lipolytica*, *Rhodotorula mucilaginosa*, *Hansenula fabiani*, *Candida parapsilosis*, *Rhodotorula rubra*, *Candida curvata*, *Enterococcus faecium*, *Rhizopus oligoporus*, *Mucor circinelloides*, *Aureobasidium pullulans, Cladosporium oxysporum*, *Clavispora lusitaniae*, *Candida intermedia*, *Lodderomyces elongisporus*, *Candida sake*, and *Candida tropicalis*.
Douchi	A traditional fermented black soybean product	China	Starter Culture (*Actinomucor elegan*)	*Lactobacillus*, *Weissella*, *Pediococcus*, *Staphylococcus*, *Enterococcus*, and *Bacillus*

*Note*: Kubo et al. (2011), Rezac et al. (2018), Li et al. (2018).

## 5 NUTRITIONAL PROFILE

Miso is formed by mixing soybeans, malted rice, wheat, or barley, and salt, and then fermenting and maturing the mixture. Soybeans, the primary ingredient, contain a lot of soybean‐specific proteins (glycinin and conglycinin), polyunsaturated fatty acid‐rich lipids, vitamin E, lecithin, saponin, and isoflavones (genistein, daidzein, daidzin, and glycitein). As a result, soybeans are a functional food with a high nutritive value, including amino acids and isoflavones, due to fermentation (Okouchi et al., 2019). Soybeans have a high protein content, and their peptide profiles/sequences offer nutritional benefits to consumers. When these proteins are hydrolyzed, a set of short‐chain peptides with 3–10 amino acids are released, which have a variety of health‐promoting properties, such as antihypertensive properties (Varnosfaderani et al., 2019).

Interest in functional foods, particularly fermented foods, has grown in recent years due to the bacteria found in them, which provide additional health advantages, nutrition, and the potential to fight/resist certain diseases. Furthermore, because humans are now attempting to investigate things beyond Earth's protective bounds, fermented foods may be used as a diet in space for astronomers due to their nutritional properties. For decades, people have been eating a wide variety of fermented foods as part of their diets. Advanced topics such as food technology, microbiology, and nutrition are investigating fermented foods and providing evidence that we should eat fermented foods more and more in the future for a healthy body (C Borresen et al., 2012).

A protein found in miso‐fermented soy paste neutralizes lipopolysaccharide (LPS), a bacterial product produced by *Escherichia coli* that promotes intestinal inflammation. Miso and its protein are believed to be used as a probiotic in humans and domestic animals to combat intestinal inflammation. As it is an LPS‐neutralizing agent, miso is useful not only as a food seasoning but also as a functional food (Sasaki et al., 2020). Many researchers recommend taking miso soup daily. Miso's health advantages have received worldwide attention, and research is being conducted in a variety of institutes to provide more experimental evidence on the benefits of miso consumption. Miso can be preserved and consumed for several years, and it retains its yellow‐brown color and aroma during that time. Table 2 shows the nutritional content and composition of miso.

**TABLE 2:** Nutritional composition of the Soyabean miso

Nutritional substances g/100 g Soybean miso (g)	
Protein	17
Fat	11
Carbohydrate	15
Ash	13
Salt	12
Iron	0.004
Calcium	0.15
Sodium	4.3
Water	45
Vitamins & vitamin precursors	
Tocopherols	0.0024
Vitamin B_6_	0.00013

*Note*: Minamiyama and Okada (2003).

## 6 THERAPEUTIC POTENTIAL

Traditional probiotics have long been a part of the diet in various cultures around the world. Fermented milk, yogurt, cheese, ice cream, curd, fruit juices, and drinks have been consumed for a long time and are being promoted due to their potential health advantages. It is widely acknowledged that the Japanese diet and food preparation practices have played a significant influence on the Japanese people's long life. Soybeans and their fermented products include a variety of functional components, including soy proteins and isoflavones, that are believed to provide therapeutic effects (Jayachandran & Xu, 2019). Scientific evidence backs up miso's extraordinary medicinal qualities. An epidemiology study in Japan discovered that persons who consume miso soup daily have a lower risk of stomach cancer and heart disease (Yamamoto et al., 2003). Fermented soy foods, such as miso, have antidiabetic, antioxidative, anti‐inflammatory, anticancer, and antihypertensive properties (Jayachandran & Xu, 2019). Various potential therapeutical properties of the miso are described in this review. Table 3 shows different important functions of soy components and their molecular mechanisms.

**TABLE 3:** Different important functions of soy components and their molecular mechanisms

S. no.	Soy component	Application	Molecular mechanism	References
1.	Bioactive peptides	Antihypersensitive, antioxidative, antidiabetic, anticancerous, antiobesity, and immunostimulatory.	Act as competitive inhibitors of enzymes involved in diabetes and cholesterol production (dipeptidyl peptidase IV, HMG Co‐A reductase, and others).	Endres (2001)
2.	Saponins	Anti‐inflammatory, cardioprotective effects, antimicrobial, anticarcinogenic.	Form complexes with cholesterol, preventing it from being absorbed in the gut, as well as inhibiting tumor‐associated enzymes and hormone receptors.	Chatterjee et al. (2018)
3.	Protease inhibitors	Antiproliferative	Chymase, trypsin, chymotrypsin, and mitogen‐triggered protein kinase activities are all inhibited. Protease activities are also downregulated, which play a key role in cancer.	Srikanth & Chen (2016)
4.	Isoflavones	Anticancerous, antiestrogen, antifibrosis, osteoporosis, type 2 diabetes, antiatherosclerosis, neuroprotection, antioxidant, etc.	Because they have structural similarities to estrogens, they form complexes with ER receptors and modulate estrogen receptor signaling pathways.	Srikanth & Chen (2016)
5.	Soy isoflavone and its metabolite	Antidiabetic	Decreased risk of type 2 diabetes	Ko et al. (2015)
6.	Soy phytochemical extract	Antidiabetic	Inhibition of intestinal glucose uptake and protection against glucose‐induced oxidation	Garg et al. (2016)

*Note*: Dukariya et al. (2020).

## 7 GASTROINTESTINAL EFFECTS

The effect of miso consumption on gastrointestinal diseases has been poorly understood. According to research, persons who consume miso soup daily have a lower risk of developing stomach illnesses such as gastritis, gastric, and duodenal ulcers than those who consume it infrequently or never (Mano et al., 2018). Another survey that elaborated on the eating behaviors of people by age showed a reduced risk of stomach diseases in those who eat miso every day in their 60s and older (Watanabe, 2013). Lately, bacteria involved in stomach inflammation and peptic ulcers, *Helicobacter pylori*, have been identified, and it is also related to stomach cancer (Brenner et al., 2009). Isoflavones present in miso include genistein; having an inhibitory effect on tyrosine kinase and particularly exhibited a potent anti‐*H. pylori* activity (Minamiyama & Okada, 2003). During the fermentation process of miso, enzymes and microorganisms destroy a substantial part of the proteins found in soybeans. In addition, miso contains a large number of highly active enzymes that aid in the digestion and absorption of other vital nutrients. Miso also contains plant fibers that are thought to be involved in “clean the intestines” (Minamiyama & Okada, 2003). Miso also has microbes that antagonize putrefactive bacteria in the intestines and have an important role in decomposing harmful compounds in the body (Marco et al., 2017). More extensive research is required to evaluate the beneficial effects of miso consumption in the reduction or inhibition of gastrointestinal diseases.

## 8 ANTICANCER POTENTIAL

Cancer is a condition in which aberrant cells divide uncontrolled and spread to other regions of the body, a process known as metastasis. Cancer can be prevented in 30%–40% of cases by adopting a healthy lifestyle and eating the right foods. Numerous high‐nutrient food components can serve as anticancer molecules, assisting in the prevention of a variety of cancers. Various studies have reported that fermented soy product consumption could be beneficial in the reduction in cancer risk. The results of a study showed that fermented soy milk drinks reduced the in vitro proliferation of the human colon cancer cell lines HT‐29 and Caco‐2 (Kim et al., 2021). Fermented soy milk inhibits the development of estrogen‐receptor‐positive cells by suppressing the production of reactive oxygen species (Zhu et al., 2006). The fermented soy milk beverage suppressed mammary carcinogenesis in rats; the soy milk beverage was fermented utilizing beneficial *Bifidobacterium*, which encompasses the higher amounts of isoflavones in soy milk.

Breast cancer is the deadliest neoplasm in women all over the world, and it causes significant health problems. Seo et al., 2009 discovered that chungkookjang, a fermented soy product from Korea, had anticancer properties against human stomach adenocarcinoma cells in research. *B. subtilis*, *B. amyloliquefaciens*, and *B. cereus* ferment soybeans for a few days to make chungkookjang. Chungkookjang inhibited apoptosis and boosted Bax while decreasing bcl‐2 mRNA expression, according to their findings. Hepatocellular carcinoma (HCC) is a disease with a wide range of molecular and clinical characteristics. The progression of HCC is linked to some factors, including viral infections and changes in the host's genetic expression (Jayachandran et al., 2015). HCC can also be prevented by using miso, according to recent research. Miso has an effect on HCC prevention, which might be due to hormonal changes, but it also lowers cell proliferation and could have a direct effect on angiogenesis and tumorous cells (Sharp et al., 2005).

## 9 ELIMINATION OF RADIOACTIVE EFFECTS

After the Chornobyl nuclear power plant catastrophe in 1986, miso exports to European nations rose due to the belief that miso consumption reduces body radiation exposure (Newmark et al., 2009). Researchers studying microorganism activity discovered that consuming miso aids in the removal of radioactive components from the body (Watanabe, 2013). Individuals working at the miso plant were less harmed by radiation than other people following the atomic bombings of Hiroshima and Nagasaki after World War II. The cause for miso's radiation‐protective properties is yet unknown. However, results from certain experiments revealed that rats fed miso were able to eliminate radioactive components from their bodies faster than animals who were not fed miso (Minamiyama & Okada, 2003).

## 10 ANTICHOLESTEROLEMIC AND ANTIAGING

Consumption of soy foods has been shown to lower blood cholesterol, thus lowering the risk of heart disease. Tofu is cholesterol free and typically has minimal saturated fats, which protects against coronary artery disease. Miso also has vital compounds, including plant sterols, linoleic acid, and vitamin E, along with some others, has an important role as cardioprotective. Substituting soy protein in place of animal protein or directly including soy protein in normal day diets/meals has clinically and experimentally proven to lower blood cholesterol levels, irrespective of the type or quantity of fat in the food (Cardoso Umbelino Cavallini et al., 2016). For example, a study was done on 15 healthy nonvegetarian premenopausal women, who showed a decrease in total blood cholesterol by taking 50 g miso/day (45 mg conjugated isoflavones) followed‐up over 9 months (Minamiyama & Okada, 2003).

Scientists are in search of finding any relationship between soy products with osteoporosis and renal disease. In a recent study, middle‐aged and elderly Japanese individuals showed an inverse connection between miso soup consumption and heart rate. The participants who said they ate miso soup regularly had a lower heart rate (Kokubo et al., 2007).

## 11 HYPERTENSION PERSPECTIVES

The previous research stated hypertension people with stage 1 or more tension people taking two miso soup servings up to 3 months every day, did not show any effect on blood pressure levels (Ito, 2020; Kondo et al., 2019). Moreover, miso is also noticed to decrease the incidence of cardiovascular diseases (CVD) by a community‐based prospective study (Kokubo et al., 2013). It was also noticed by the survey that Japanese people consuming fermented soy products, like natto and miso, had a negative relation with the progress of high blood pressure in normotension adults (Nozue et al., 2017). Miso intake does not enhance hypertension or high blood pressure levels in those who have normotension or mild hypertension issues, according to these trials and research.

The relationship between miso soup intake and blood pressure and heart rate was investigated in four groups of Japanese individuals, each defined by a different season. People who do not consume miso soup regularly have been found to have higher heart rates in the winter than in any other season. People who consume a lot of miso soup, on the other hand, do not have a higher heart rate in the winter than in other seasons. Because autonomic nerve balance is linked to sympathetic nerve activity (SNA) and plays a role in regulating heart rate, heart rate is utilized as a biomarker of SNA. As a result, to minimize the risk of cardiovascular disease by reducing SNA levels, miso soup consumption should be increased throughout the winter season (Nozue et al., 2017).

## 12 ANTIOBESITY

Obesity is the world's most serious health problem, and it has reached epidemic proportions. New targets have been established to recognize the molecules that govern the distribution, organization, and breakdown of adipose tissue, which will aid in the prevention and treatment of obesity. Food science and nutrition research has revealed the possibility of utilizing food‐derived components to balance specific physiological processes and molecular signaling in humans, to regulate and delay obesity growth at the molecular level (Pan et al., 2016). Soy meals are high in isoflavones, which may work in conjunction with intracellular estrogen receptors to reduce fat formation and adipose tissue distribution. Soy meals and their components have been demonstrated to have antiobesity properties in several studies. Soy isoflavones and their derivatives have a structural similarity to 17‐estradiol (E2) and have been shown to have an estrogenic action with estrogen‐receptor‐binding affinity. Estrogen receptors may be present in several cells and organs, including adipose tissues, where they are important for metabolism and fat distribution (Pallottini et al., 2008).

Hypertrophy (increased adipocyte size) and hyperplasia (increased adipocyte quantity) are two key features of adipose tissue that contribute to obesity (increased adipocyte number). Both of these processes have been demonstrated to be reduced by soy isoflavones. In mice, genistein has been shown to diminish adipose tissue in vivo by lowering adipocyte counts, whereas daidzein has been shown to reduce total fat mass in vivo by reducing adipocyte numbers. Several studies have shown that soy protein and peptides are the active ingredients in lowering LDL cholesterol and triacylglycerol in the human body (Kim et al., 2011).

## 13 CONCLUSION

Miso, in particular, is an important part of the Japanese diet because it contains a wide variety of nutritional fermentation products derived from soybeans and grains. Miso possesses anticancer, antihypertensive, antiobese, and anti‐inflammatory properties, as well as the ability to eliminate gastrointestinal diseases. To ensure more production of traditional fermented products around the world, further research is needed to understand how the succession of microorganisms during the fermentation process varies depending on geographic location, starter culture use, climate, and environmental differences, both in homes and commercial production.

## CONFLICT OF INTEREST

The authors declare that they have no conflict of interest.

## CONSENT TO PARTICIPATE

Corresponding and all the coauthors are willing to participate in this manuscript.

## Data Availability

The data that support the findings of this study are available on request from the corresponding author. The data are not publicly available due to privacy or ethical restrictions.
